# Effect of the Aortic Root Infusion of Sufentanil on Ischemia-Reperfusion Injury in Patients Undergoing Coronary Artery Bypass Grafting: A Randomized Clinical Trial

**Published:** 2019-10

**Authors:** Mohammad Bagher Khosravi, Mahdi Kahrom, Mahdi Tahari, Kambiz Alizadeh, Ghasem Soltani, Mohammad Ali Ghanad

**Affiliations:** 1 *Department of Anesthesia, School of Medicine, Shiraz University of Medical Sciences, Shiraz, Iran.*; 2 *Department of Cardiovascular Surgery, School of Medicine, Mashhad University of Medical Sciences, Mashhad, Iran.*; 3 *Division of Cardiac Perfusion, * *School of Medicine,* * Shiraz University of Medical Sciences, Shiraz, Iran.*; 4 *Department of Anesthesia, School of Medicine, Mashhad University of Medical Sciences, Mashhad, Iran.*

**Keywords:** *Cardiopulmonary bypass*, *Cardiac surgical procedures*, *Sufentanil*, *Ischemic postconditioning*, *Reperfusion injury*

## Abstract

**Background: **Ischemic postconditioning is a novel strategy for attaining cardioprotection. Remarkable evidence from various in vitro and in vivo animal and human studies have shown significant opioid-induced cardioprotection against myocardial ischemia/reperfusion (I/R) injury. The purpose of this study was to assess the cardioprotective effect of sufentanil against I/R injury after on-pump coronary artery bypass grafting (CABG).

**Methods: **Between June 2016 and July 2017, 80 consecutive patients with triple-vessel disease undergoing on-pump CABG were enrolled in this prospective randomized study. The patients assigned to the sufentanil group received a single dose of sufentanil (0.2 μg/kg diluted with 50 cc of saline) 5 minutes before the removal of the aorta cross-clamp, with the sufentanil injected via a cardioplegia needle into the aortic root. In the control group, the same volume of normal saline was injected as a placebo. Cardiac enzymes, the inotrope score, and the outcome data were compared between the 2 groups.

**Results**: The mean age of the patients was 60.48±7.50 years (range=41–69 y), and men comprised 65.0% of the study population. The levels of CK-MB and cardiac troponin I were significantly lower in the sufentanil group (P<0.001). The amount of inotrope use (P<0.001), the incidence of atrial fibrillation (P=0.014), electrical shock (P=0.007), and the mechanical ventilation time (P<0.001) decreased in the sufentanil group compared with the control group. However, the use of intra-aortic balloon pumps (P=0.247) and the ICU length of stay (P=0.867) were not significantly different between the 2 groups.

**Conclusion**: The injection of a single dose of sufentanil into the aortic root prior to aorta cross-clamp removal diminished cardiac injury during on-pump CABG in our patients.

## Introduction

Ischemic/reperfusion (I/R) injury is associated with tissue damage following the reperfusion of an ischemic tissue.^1^ Postconditioning, first described by Zhao and colleagues in dogs, is a novel strategy for attaining cardioprotection to avoid I/R injury; it is defined as brief intermittent repetitive interruptions at the onset of reperfusion after a prolonged period of ischemia, reducing myocardial injury.^2^^, ^^3^ Nonetheless, at the early stages of tissue reperfusion, it is difficult to apply discontinuous episodes of myocardial I/R and intermittent cross-clamping is associated with possible complications such as left ventricular distention or aortic injury.

Such problems in clinical practice can be obviated through postconditioning, and pharmacological agents can be easily utilized to mimic ischemic myocardial postconditioning using drugs at the onset of reperfusion.^4^^, ^^5^ During pharmacological postconditioning, the drug is directly delivered to the myocardium via the aortic root to prevent drug dilution during cardiopulmonary bypass and the immediate onset of drug effects.

Various opioid drugs have been studied as pharmacological postconditioning strategies, with some having been proven to ameliorate myocardial damage when applied before, during, or shortly after myocardial I/R. The activation of the κ- and/or δ-opioid receptor is involved in direct myocardial protection, while the role of μ-opioid receptors appears less clear.^6^ Nevertheless, there is currently a dearth of data on the clinical aspects of opioid myocardial protection.^7^

Sufentanil is a synthetic opioid analgesic and is widely used for anesthesia in cardiac surgery. It is a specific μ-opioid-receptor agonist, and its powerful myocardial protection against I/R injury has been confirmed in rats.^4^

This study aimed to investigate whether sufentanil postconditioning in patients undergoing coronary artery bypass grafting (CABG) with an otherwise standardized anesthetic technique would protect the heart against I/R injury.

## Methods

Between June 2016 and July 2017, 80 consecutive patients with triple-vessel disease undergoing on-pump CABG were enrolled in this prospective randomized study. The patients were randomly assigned into either a control or sufentanil-postconditioning group by computerized block randomization method. 

The primary inclusion criterion for patient selection was the diagnosis of coronary artery disease appropriate for CABG on preoperative angiography. The exclusion criteria included opioid addiction (daily use of opium or its derivatives during the preceding 6 months), diabetes mellitus (fasting blood sugar ≥127 mg/dL), end-stage renal failure on dialysis, hepatic dysfunction, an ejection fraction of less than 35%, valvular heart diseases (need for combined valvular surgery), acute myocardial infarction, urgent or emergent surgery, previous cardiac surgery, and the need for ventricular aneurysmectomy or other surgical procedures. 

**Figure 1:**
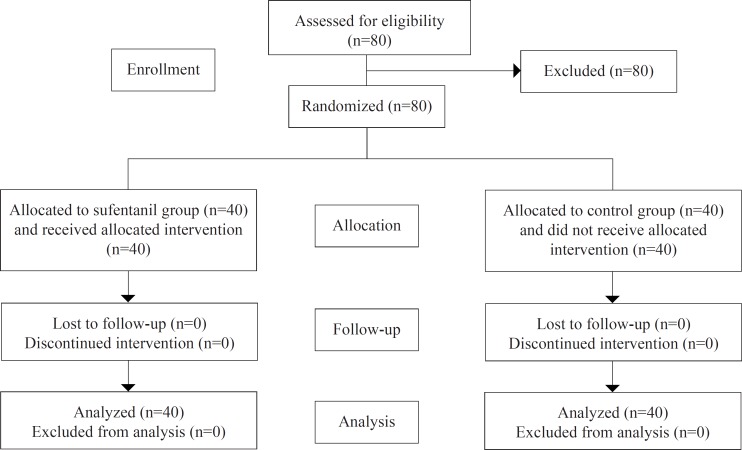
Participants’ follow-up diagram

During the surgical procedure, with the objective of equalizing the opioid effect, anesthesia was induced and maintained with a similar dose of fentanyl in both groups. The patients assigned to the sufentanil group received a single dose of sufentanil (0.2 μg/kg diluted with 50 cc of saline) 5 minutes before the removal of the aorta cross-clamp. Sufentanil was injected via a cardioplegia needle into the aortic root for a direct and focused delivery of the drug to the heart over a 1-minute period. The same strategy was adopted in the control group with the same volume of normal saline instead (Figure 1).

At the end of the surgery, the patients were transferred to the intensive care unit (ICU) and managed by cardiac anesthesiologists for inotrope and ventilatory support, hemodynamic stabilization, temperature, fluids, and electrolyte balance. The patients were extubated as soon as they met the following criteria: consciousness with pain control, acceptable respiratory force and arterial blood gas, hemodynamic stability, normothermia, and the absence of excessive bleeding. Following surgery, all the patients were followed for at least 30 days.

Prospective cohort data were collected by blind trained list reviewers using standard data forms. The elicited information covered the patients’ demographic characteristics, age, sex, risk factors, and outcome variables including low cardiac output state, reoperation for bleeding or tamponade, malignant arrhythmias, atrial fibrillation, postoperative myocardial infarction, blood transfusion, pleural effusion, sepsis or endocarditis, respiratory failure, transient ischemic attack or stroke, intra-aortic balloon pump use, postoperative echocardiographic data, ICU and hospital lengths of stay, in-hospital death, and early mortality (≤30 d). Before the induction of anesthesia and at 4, 8, and 24 hours after aorta unclamping, blood samples for CK-MB and cardiac troponin I levels were obtained from the central venous line. Based on the following formula,^8^ quantitative inotrope measurements for the first postoperative day were done: inotropic score = ([dopamine + dobutamine]×1) + (milrinone×15) + ([epinephrine + norepinephrine+ isoproterenol]×100).

The clinical data were compared using the independent *t*-, Mann–Whitney *U*, Wilcoxon Signed Ranks, and χ^2 ^tests, whenever appropriate. A P value of less than 0.05 was considered statistically significant. The statistical analyses were performed using the SPSS statistical software package, version 20 (IBM SPSS Statistics for Windows, Version 20.0. Armonk, NY: IBM Corp.).

The study protocol was approved by the institutional medico-ethical review committee, and written informed consent was signed by each patient prior to enrollment.

## Results

Between June 2016 and July 2017, 80 consecutive patients who met the mentioned inclusion criteria were randomly assigned into either a sufentanil-postconditioning group (n=40) or a control group (n=40). The mean age of the patients was 60.48±7.50 years (range =41–69 y), and men accounted for 65.0% of the study population. There were no significant differences in patient characteristics between the 2 study groups in terms of the demographic characteristics, age, gender, weight, the left ventricular ejection fraction, medications, pre-existing medical conditions, laboratory results, and risk factors (Table 1). There were also no significant differences in the duration of cardiopulmonary bypass, the aortic cross-clamp time, and other perfusion parameters.

The levels of CK-MB and cardiac troponin I were not significantly different between the groups at the induction of anesthesia but were significantly lower in the sufentanil group at 4, 8, and 24 hours after the removal of the aorta cross-clamps (P<0.001) (Table 2 and Table 3). Furthermore, the inotrope score at 12 and 24 postoperative hours was significantly lower in the sufentanil group (Table 4). Postoperative atrial fibrillation rhythms were less frequent in the sufentanil group (P=0.014). Additionally, in the sufentanil group, the time to tracheal extubation was significantly shorter (P<0.001). Nevertheless, the overall hospital and ICU lengths of stay were not different between the 2 groups (P=0.867), and nor was there any mortality in the 2 groups. Two patients in the control group developed a postoperative low cardiac output state, which subsequently required intra-aortic balloon pump support.

**Table 1:** Characteristics of the patients in the sufentanil and control groups*

	Sufentanil Group (n=40)	Control Group (n=40)	P value
Age (y)	60.02±8.46	60.48±7.50	0.791
Male gender	27 (67.5)	25 (62.5)	0.639
Height (cm)	164.25±10.02	165.38±9.45	0.607
Weight (kg)	71.08±14.29	68.65±12.00	0.414
Body surface area (m²)	1.74±0.20	1.75±0.19	0.826
Preoperative ejection fraction (%)	44.25±5.94	43.75±6.18	0.713
Hypertension	21 (52.5)	24 (60.0)	0.499
HLP	32 (80.0)	29 (72.5)	0.431
Cross-clamp time (min)	50.48±4.71	50.63±8.38	0.922
Cardiopulmonary bypass time (min)	78.72±6.70	82.01±13.15	0.165
Intubation time (h)	10.39±1.17	12.19±2.81	<0.001
ICU stay (d)	44.72±1.69	44.94±8.18	0.867

HLP, Hyperlipidemia; ICU, Intensive care unit

* Data are presented as mean±SD, n (%)

**Table 2:** Serum levels of CK-MB in the sufentanil and control groups over time*

	Sufentanil Group	Control Group	P value
CK-MB at induction time (U/L)	18.30±8.25	19.50±7.13	0.489
CK-MB 4 h after unclamping (U/L)	65.25±15.63	95.75±22.55	<0.001
CK-MB 8 h after unclamping (U/L)	45.58±13.91	73.78±18.77	<0.001
CK-MB 24 h after unclamping (U/L)	27.83±11.45	41.63±12.51	<0.001

* Data are presented as mean±SD

**Table 3:** Serum levels of troponin in the sufentanil and control groups over time*

	Sufentanil Group	Control Group	P value
Troponin at induction time (µg/L)	0.10±0.28	0.17±0.33	0.468
Troponin 4 h after unclamping (µg/L)	1.73±0.79	3.24±1.29	<0.001
Troponin 8 h after unclamping (µg/L)	1.26±0.68	2.49±1.18	<0.001
Troponin 24 h after unclamping (µg/L)	0.77±0.55	1.23±0.66	<0.001

* Data are presented as mean±SD

**Table 4:** Comparison of the inotrope score between the sufentanil and control groups over time*

	Sufentanil Group	Control Group	Control Group
Inotrope score (during 12 h after surgery)	2.12±1.96	5.11±5.27	5.11±5.27
Inotrope score (during 24 h after surgery)	1.13±1.17	3.02±3.75	3.02±3.75

* Data are presented as mean±SD

## Discussion

Although cardioplegic arrest is a useful method for myocardial protection, it cannot completely prevent myocardial I/R injury during cardiac surgery. Encouraging results from various studies on ischemic postconditioning in humans and animals are available.^9^^-^^13^

Results from different in vivo and in vitro human studies support a role for exogenously administered and/or endogenously produced opioids in myocardial protection against I/R injury. Their physiological and pharmacological effects are due to their ability to bind to specific opioid receptors: δ, κ, and μ.^14^ Additionally, opioid peptides have been proven to exert obvious effects on cardiac muscle function in rat ventricular cardiomyocytes mediated by κ- and δ-receptors, but not μ-receptor stimulation.^15^ In clinical practice, opioids such as morphine, fentanyl, remifentanil, and sufentanil have different affinities to receptor subtypes, rendering conclusions regarding subtype involvement in opioid-mediated cardioprotection challenging. Other factors that may alter opioid-induced cardioprotection include the dose, the route of administration, and timing in relation to myocardial I/R.

Different cohorts have demonstrated that opioids can afford cardioprotection against I/R injury in animals and humans. In an investigation on children with the tetralogy of Fallot undergoing total correction, an aortic root infusion of morphine was associated with improved cardioprotection against I/R injury.^9^ In another study, Chen et al.^3^ showed that the administration of morphine at the beginning of reperfusion decreased the infarct size and the release of CK-MB in isolated rat hearts. In a small clinical trial, troponin I was markedly reduced up to 2 days postoperatively following a 5-μg/kg remifentanil bolus over 10 minutes before off-pump CABG.^16^ Similarly, in a study involving 40 patients undergoing on-pump CABG, 1 μg/kg of remifentanil bolus, followed by a 0.5-μg/kg/min infusion for 30 minutes after anesthetic induction, but before sternotomy, resulted in lower troponin I and CK-MB levels for 12 and 24 hours, respectively.^7^ Another study revealed that sufentanil, administered through the aortic root before aortic unclamping, significantly decreased postoperative inotrope requirements and the release of cardiac troponin I and CK-MB in adult patients undergoing mitral valve replacement.^4^

Sufentanil, first synthesized in 1974, is an opioid about 5 to 10 times more potent as an analgesic than fentanyl, and yet has a shorter duration of action. It is a highly selective μ-opioid-receptor agonist and produces more stable hemodynamics. Wu and colleagues^17^ showed that modulating Bax and Bcl-2 expression and activating the PI3K/Akt-GSK-3β pathway during sufentanil postconditioning lessened myocardial injury. 

Current in vitro and in vivo evidence indicates that sufentanil may produce important effects to protect the myocardium against I/R injury and represent promising approaches to decreasing I/R injury; still, additional large scale clinical trials to validate the early findings are needed. Our study showed that an injection of a single dose of sufentanil (0.2 μg/kg diluted with 50 cc of saline) via a cardioplegia needle into the aortic root 5 minutes before aorta cross-clamp removal attenuated the release of CK-MB and cardiac troponin I and reduced postoperative inotrope requirements in adults undergoing elective CABG. Postoperative atrial fibrillation rhythms and the time to tracheal extubation were also significantly lower in the patients in the sufentanil group. However, we used a similar dosage of opioid agents for the induction of anesthesia and maintenance in both groups to neutralize the possible cardioprotective effect of different opioids.

In an animal study, sufentanil postconditioning significantly diminished the size of myocardial infarct by 46% in nondiabetic rats (P<0.001), but diabetes prevented the cardioprotective effect of sufentanil, which was restored during long-term insulin treatment.^18^

In the present study, we excluded diabetic patients because diabetes ameliorated the cardioprotective effect of sufentanil postconditioning in an animal study.^19^^, ^^20^ The activity of GSK-3β is increased in diabetes mellitus, which induces the permeability of mitochondrial transition pore in rats.

The limitations of this study were its short-term period of postoperative follow-up (30 days after the operations) and the use of only cardiac enzymes and echocardiographic indices for the evaluation of myocardial injury and left ventricular function.

## Conclusion

Our study revealed that the injection of a single dose of sufentanil into the aortic root prior to aorta cross-clamp removal diminished the release of cardiac biochemical markers and myocardial injury during on-pump CABG and is a safe and effective strategy for myocardial postconditioning. To our knowledge, this study is the first clinical randomized controlled trial to assess the efficacy of sufentanil postconditioning on cardiac protection in patients undergoing CABG.

## Notes:

***This paper should be cited as:*** Bagher Khosravi M, Kahrom M, Tahari M, Alizadeh K, Soltani G, Ghanad MA. Effect of the Aortic Root Infusion of Sufentanil on Ischemia-Reperfusion Injury in Patients Undergoing Coronary Artery Bypass Grafting: A Randomized Clinical Trial. J Teh Univ Heart Ctr 2019;14(4):177-182.
